# Treatment patterns and outcomes of older patients with mantle cell lymphoma in an Asian population

**DOI:** 10.1186/s12885-021-08326-1

**Published:** 2021-05-17

**Authors:** Xinyi Yang, Lay Poh Khoo, Esther Wei Yin Chang, Valerie Shiwen Yang, Eileen Poon, Nagavalli Somasundaram, Mohamad Farid, Tiffany Pooi Ling Tang, Miriam Tao, Soon Thye Lim, Jason Yongsheng Chan

**Affiliations:** 1 Yong Loo Lin School of Medicine, National University of Singapore, Singapore City, Singapore; 2 Division of Medical Oncology, National Cancer Centre Singapore, 11 Hospital Drive, Singapore City, 169610 Singapore; 3 SingHealth Duke-NUS Blood Cancer Centre, Singapore City, Singapore; 4 Institute of Molecular and Cell Biology, Singapore City, Singapore; 5 Oncology Academic Clinical Program, Duke-NUS Medical School, Singapore City, Singapore; 6 Cancer Science Institute of Singapore, National University of Singapore, Singapore City, Singapore

**Keywords:** Prognostic biomarker, Cytarabine, Non-Hodgkin lymphoma, Chemotherapy

## Abstract

**Background:**

Significant progress has been made in the treatment outcomes of mantle cell lymphoma (MCL) since the introduction of cytarabine and rituximab in modern regimens. However, older patients may not readily tolerate these agents nor derive benefit. We investigated the impact of age on treatment patterns and clinical outcomes of MCL patients in an Asian population.

**Methods:**

A retrospective study was conducted on patients (*n* = 66) diagnosed with MCL at the National Cancer Centre Singapore between 1998 and 2018. The median follow-up duration was 40 months. Survival analyses were performed using the Kaplan-Meier method and multivariate Cox proportional models.

**Results:**

The median age of the cohort was 59 years (range, 26–84), with a male predominance (73%). The majority (86%) had advanced stage 3–4 disease at diagnosis. Compared with younger patients, older patients aged ≥60 years (*n* = 32; 48.5%) presented more frequently with B-symptoms (75% vs 38%, *p* = 0.0028), anaemia (75% vs 35%, *p* = 0.0013), and carried higher prognostic risk scores (sMIPI high risk 84% vs 56%, *p* = 0.016). Non-cytarabine-based induction chemotherapy was more commonly administered in older patients (76% vs 32%, *p* = 0.0012). The 5-year overall survival (OS) and progression-free survival (PFS) was 68 and 25% respectively. In a multivariable model, older age (HR 3.42, 95%CI 1.48–7.92, *p* = 0.004) and anemia (HR 2.56, 95%CI 1.10–5.96, *p* = 0.029) were independently associated with poorer OS while older age (HR 2.24, 95%CI 1.21–4.14, *p* = 0.010) and hypoalbuminemia (HR 2.20, 95%CI 1.17–4.13, *p* = 0.014) were independently associated with poorer PFS. In an exploratory analysis, maintenance rituximab following induction chemotherapy improved PFS in younger patients, with median PFS of 131 months and 45 months with or without maintenance therapy respectively (HR 0.39, 95%CI 0.16–0.93, *p* = 0.035). In contrast, no survival benefit was observed in older patients.

**Conclusions:**

We demonstrated in our analysis that older patients with MCL may harbor adverse clinical features and may not derive benefit from maintenance rituximab, highlighting the need for further research in this area of need.

**Supplementary Information:**

The online version contains supplementary material available at 10.1186/s12885-021-08326-1.

## Introduction

Mantle cell lymphoma (MCL) is a rare B cell non-Hodgkin lymphoma characterized by distinct genetic alterations and immunophenotype [1]. Clinically, majority of MCL cases occur in the male gender and older patients [2]. Although it is recognized that the clinical course and treatment responses often exhibit significant heterogeneity, this disease entity is typically aggressive and portends a poor prognosis [3].

Contemporary treatment for MCL constitutes a choice of various induction chemotherapy regimens followed by a consideration for autologous stem cell transplant (auto-SCT) and/or maintenance rituximab (MR), with the ultimate decision depending largely on patient factors as well as disease biology. For young and fit patients, the administration of intensive rituximab-based immuno-chemotherapy regimens incorporating high-dose cytarabine with or without auto-SCT are accepted first line treatment options [4–7]. The management of the older patient with MCL remains highly challenging, as the majority these patients are not candidates for such intensive treatment regimens. Alternative less-intensive induction strategies, including R-CHOP and R-bendamustine are generally preferred for this group of patients provided they are not frail [2]. The use of MR remains an option, though the derived benefit may depend on the initial choice of induction therapy [8, 9].

In this study, we investigate the clinical outcomes of MCL patients in an Asian population and examine the impact of age on their treatment patterns and clinical outcomes.

## Patients and methods

### Study cohort

Patients who were diagnosed with MCL and seen at the National Cancer Centre Singapore between April 1998 and June 2018 were retrospectively analysed. A total of 66 patients were included in the final analysis. The median follow-up duration was 39.6 months. Relevant demographical, clinico-pathological and haematological information were collected and utilized for the analysis. Demographical information included sex, age, ethnicity and smoking history. Age, sex, and ethnicity of the patients were corroborated against their National Registry Identification Cards. Clinical characteristics included the presence of B-symptoms, Eastern Cooperative Oncology Group (ECOG) performance status, Ann Arbor staging, sites and bulk of disease, as well as simplified MIPI risk scores (sMIPI). Haematological characteristics included peripheral blood haemoglobin (Hb) and leucocyte (WBC) counts, serum lactate dehydrogenase (LDH) levels and serum albumin levels. Treatment information was also collected for analysis, including the choice of first line-chemotherapy, use of MR, and conduct of autologous stem cell transplantation.

All data were obtained at the time of diagnosis or subsequent follow-up. Written informed consent for use of biospecimens and clinical data were obtained in accordance with the Declaration of Helsinki. The research study was carried out as part of the Singapore Lymphoma Study with approval from the SingHealth Centralised Institutional Review Board (CIRB 2018/3084). Participants and/or their legal guardians provided informed consent for their data to be used in this research. The datasets created and analysed during this study are available from the corresponding authors upon reasonable request.

### Statistical analysis

The primary outcomes of this study are overall survival (OS) and progression-free survival (PFS). OS was calculated from the date of diagnosis up to the date of death from any cause or was censored at the date of last follow-up for survivors. PFS was defined as the time elapsed between the date of diagnosis to the date of relapse, progression, or death from any cause. Kaplan-Meier survival curves were plotted to estimate survival for each individual clinico-pathological parameter. The log-rank test was then used to determine hazard ratios (HR), the corresponding 95% confidence intervals (95% CI) of mortality and the *p*-values. Subsequently, parameters with significance level of < 0.05 were used in the generation of multivariable Cox regression models via a backward regression approach to test for independence of significant factors. Comparisons of the frequencies of categorical variables were performed using Pearson’s Chi-squared test or Fisher’s exact test, as appropriate. All statistical evaluations were made assuming a two-sided test with significance level of 0.05 unless otherwise stated. All tests were performed using MedCalc statistical Software for Windows version 19.0.4 (MedCalc Software, Ostend, Belgium).

## Results

### Patient demographics

A total of 66 patients were included in the study. The median age of diagnosis was 59 years (range: 26 to 84 years). Forty-eight (72.7%) were male and 18 (27.3%) were female. In terms of initial staging, 9 (18.2%) patients were Ann-Arbor stage 1–2 at diagnosis while 57 (86.4%) were stage 3–4; 41 (62.1%) had bone marrow involvement. Twenty (30.3%) patients were classified as low risk, 30 (45.5%) as intermediate risk, and 16 (24.2%) as high risk by the sMIPI prognostic index. Clinical and demographic characteristics of all patients are summarized in Table 1.

**Table 1:** Clinical and demographic characteristics of patients with MCL in our cohort

Characteristic	***N*** (%)
***Total***	66 (100)
***Age (years)***	
**Median (range)**	59 (26 to 84)
**≥ 60**	32 (48.5)
**< 60**	34 (51.5)
***Sex***	
**Male**	48 (72.7)
**Female**	18 (27.3)
***Ethnicity***	
**Chinese**	50 (75.8)
**Malay**	7 (10.6)
**Indian**	3 (4.55)
**Others**	6 (13.6)
***Smoking history***	
**Yes**	9 (13.6)
**No**	57 (86.4)
***B-symptoms***	
**Absent**	45 (68.2)
**Present**	21 (31.8)
***ECOG performance status***	
**0**	40 (60.6)
**1–4**	26 (39.4)
***Ann Arbor stage***	
**1–2**	9 (18.2)
**3–4**	57 (86.4)
***sMIPI risk***	
**Low**	20 (30.3)
**Intermediate**	30 (45.5)
**High**	16 (24.2)
***Bulky disease > 10 cm***	
**Yes**	6 (9.5)
**No**	57 (90.5)
***Spleen involved***	
**Yes**	20 (30.3)
**No**	46 (69.7)
***Number of nodal sites***	
**0–3**	26 (39.4)
**≥ 4**	40 (60.6)
***Extra-nodal involvement***	
**Yes**	54 (81.8)
**No**	12 (18.2)
***Bone marrow involvement***	
**Positive**	41 (62.1)
**Negative**	25 (37.9)
***Ki-67 expression (%)***	
**> 30**	22 (47.8)
**≤ 30**	24 (52.2)
***Serum LDH***	
**Elevated**	35 (54.7)
**Not elevated**	29 (45.3)
***Albumin (g/L)***	
**< 35**	18 (27.7)
**≥ 35**	47 (72.3)
***Hemoglobin (g/dL)***	
**≤ 12.4**	36 (54.5)
**> 12.4**	30 (45.5)
***WBC (10***^***9***^ ***cells/L)***	
**> 10**	19 (28.8)
**≤ 10**	47 (71.2)

*Abbreviations*: *ECOG* Eastern Cooperative Oncology Group, *MIPI* Mantle Cell International Prognostic Index, *LDH* lactate dehydrogenase, *WBC* white blood cell count

Variables unknown include: presence of bulky disease > 10 cm (*n* = 3), Ki-67 expression (*n* = 20), serum albumin (*n* = 1), serum LDH (*n* = 2)

Patients were analysed by age groups (Table 2). There were 32 (48.5%) and 34 (51.5%) patients with age ≥ 60 years and age <  60 years, respectively. Compared with younger patients, older patients (age ≥ 60) presented more frequently with B-symptoms at diagnosis (75% vs 38.2%, *p* = 0.0028). A higher proportion of these older patients were classified as sMIPI high risk (84.4% vs 55.9%, *p* = 0.016), and anaemia was significantly more prevalent (75% vs 35.3%, *p* = 0.0013). Older patients tended to have poorer performance status as well, though this was not statistically significant.

**Table 2:** Association of age at diagnosis with clinical characteristics

Characteristic	Age (years)		***p***-value
	**≥ 60**	**< 60**	
***Total***	32 (48.5%)	34 (51.5%)	–
***Sex***			
**Male** (48)	24 (75%)	24 (70.6%)	0.6898
**Female** (18)	8 (25%)	10 (29.4%)	
**Ethnicity**			
**Chinese** (50)	23 (71.9%)	27 (79.4%)	0.4786
**Other** (16)	9 (28.1%)	7 (20.6%)	
***Smoking history***			
**Yes** (9)	4 (12.5%)	5 (14.7%)	1.0000
**No** (57)	28 (87.5%)	29 (85.3%)	
***B-symptoms***			
**Absent** (29)	8 (25%)	21 (61.8%)	**0.0028**
**Present** (37)	24 (75%)	13 (38.2%)	
***ECOG performance status***			
**0** (40)	16 (50%)	24 (70.6%)	0.0896
**1–4** (26)	16 (50%)	10 (29.4%)	
***Ann Arbor stage***			
**1–2** (9)	4 (12.5%)	5 (14.7%)	1.0000
**3–4** (57)	28 (87.5%)	29 (85.3%)	
***sMIPI risk***			
**Low/intermediate** (20)	5 (15.6%)	15 (44.1%)	**0.0161**
**High** (46)	27 (84.4%)	19 (55.9%)	
***Bulky disease > 10 cm***			
**Yes** (6)	3 (9.38%)	3 (8.82%)	1.0000
**No** (57)	28 (87.5%)	29 (85.3%)	
***Spleen involved***			
**Yes** (20)	10 (31.3%)	10 (29.4%)	0.8720
**No** (46)	22 (68.8%)	24 (70.6%)	
***Number of nodal sites***			
**0–3** (26)	11 (34.4%)	15 (44.1%)	0.4217
**≥ 4** (40)	21 (65.6%)	19 (55.9%)	
***Extra-nodal involvement***			
**Yes** (54)	24 (75%)	30 (88.2%)	0.2095
**No** (12)	8 (25%)	4 (11.8%)	
***Bone marrow involvement***			
**Positive** (41)	17 (53.1%)	24 (70.6%)	0.1469
**Negative** (25)	15 (46.9%)	10 (29.4%)	
***Ki-67 expression (%)***			
**> 30** (22)	12 (37.5%)	10 (29.4%)	0.5593
**≤ 30** (24)	11 (34.4%)	13 (38.2%)	
***Serum LDH***			
**Elevated** (35)	18 (56.3%)	17 (50%)	0.4262
**Not elevated** (29)	12 (37.5%)	17 (50%)	
***Albumin (g/L)***			
**< 35** (18)	11 (34.4%)	7 (20.6%)	0.1835
**≥ 35** (47)	20 (62.5%)	27 (79.4%)	
***Hemoglobin (g/dL)***			
**≤ 12.4** (36)	24 (75%)	12 (35.3%)	**0.0013**
**> 12.4** (30)	8 (25%)	22 (64.7%)	
***WBC (10***^***9***^ ***cells/L)***			
**> 10** (19)	10 (31.3%)	9 (26.5%)	0.6706
**≤ 10** (47)	22 (68.8%)	25 (73.5%)	

Variables unknown include: presence of bulky disease > 10 cm (*n* = 3), Ki-67 expression (n = 20), serum albumin (*n* = 1), serum LDH (*n* = 2)

### Treatment patterns and outcomes

Fifty-six patients (84.8%) received chemotherapy as first-line treatment, amongst which 25 (44.6%) were age ≥ 60 years and 31 (55.4%) were age <  60 years. In this subgroup analysis, older patients were also more commonly in the sMIPI high risk group (84% vs 54.8%, *p* = 0.024) and more frequently anaemic (68% vs 35.5%, *p* = 0.017) (Table S1).

In terms of chemotherapy regimens (Table 3), 27 (48.2%) received cytarabine-based regimens with a complete response rate of 81.5% and partial response rate of 18.5%. Twenty-nine patients (51.8%) received non-cytarabine-based chemotherapy, achieving a complete response rate of 65.5% and partial response rate of 27.5%. Cytarabine-based induction chemotherapy was more commonly administered in younger compared to older patients (67.7% vs 24%, *p* = 0.0012). The most frequently prescribed cytarabine-based and non-cytarabine-based regimens were R-HyperCVAD (59.3%) and R-CHOP (24.2%) respectively. Five (7.6%) patients were consolidated with auto-SCT following complete response to induction chemotherapy. Out of 56 patients who underwent chemotherapy, 25 (44.6%) received MR. Eleven patients (44%) were age ≥ 60 years and 14 (56%) were age < 60 years. Most patients receiving MR followed cytarabine-based or R-CHOP induction (age < 60 years: 10 of 14; age ≥ 60 years: 8 of 11).

**Table 3:** First line chemotherapy and response

Treatment modality	***N*** (%)		
		**Response to chemotherapy - n (%)**	
		**PR**	**CR**
**Cytarabine-based**	27 (40.9)	**5 (18.5)**	**22 (81.5)**
**R-CHOP/R-ARAC**	6 (9.1)	2 (33.3)	4 (66.7)
**R-CHOP/R-DHAP**	1 (1.5)	–	1 (100)
**R-HyperCVAD**	16 (59.3)	1 (6.25)	15 (93.8)
**HyperCVAD**	2 (3.0)	1 (50)	1 (50)
**R-BAC**	2 (3.0)	1 (50)	1 (50)
**Others**	29 (51.8)	**8 (27.5)**	**19 (65.5)**
**R-Bendamustine**	7 (10.6)	2 (28.6)	5 (71.4)
**VRCAP**	3 (4.5)	–	3 (100)
**R-CHOP**^**a**^	16 (24.2)	4 (25)	10 (62.5)
**R-CVP**	1 (1.5)	1 (100)	–
**CHOP**	2 (3.0)	1 (50)	1 (50)
***Maintenance rituximab***			
**Yes**	25 (44.6)		
**No**	31 (55.4)		
***Autologous stem cell transplant***			
**Yes**	5 (7.6)		
**No**	61 (92.4)		
***Non-chemotherapy***			
**Watch and wait only**	1 (1.8)		
**Radiation only**	3 (4.6)		
**Best Supportive Care**	3 (4.6)		
**Unknown**	3 (4.6)		

^**a**^Responses were unknown for 2 patients

### Survival analyses and prognostic factors

**Fig. 1:**
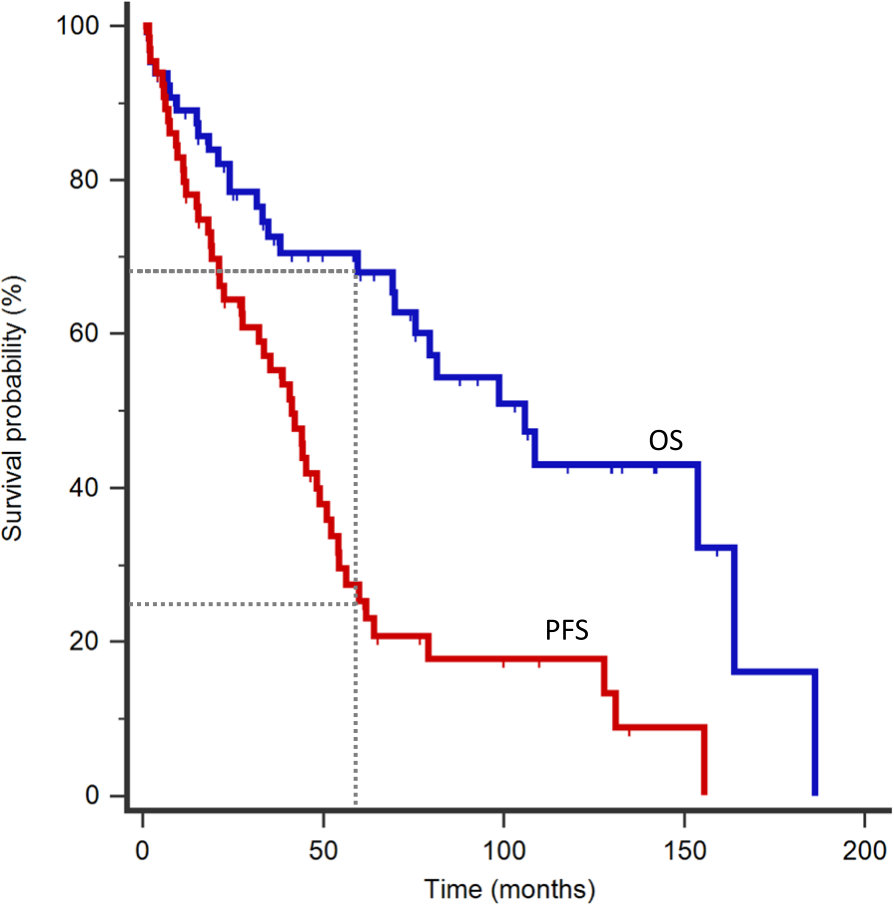
Overall survival outcomes for patients with MCL. **a** In the overall cohort (*n* = 66), 68% of the patients were alive and 25.3% were progression-free at 5 years. Median OS and PFS were 105.7 months and 41.1 months, respectively

**Fig. 2:**
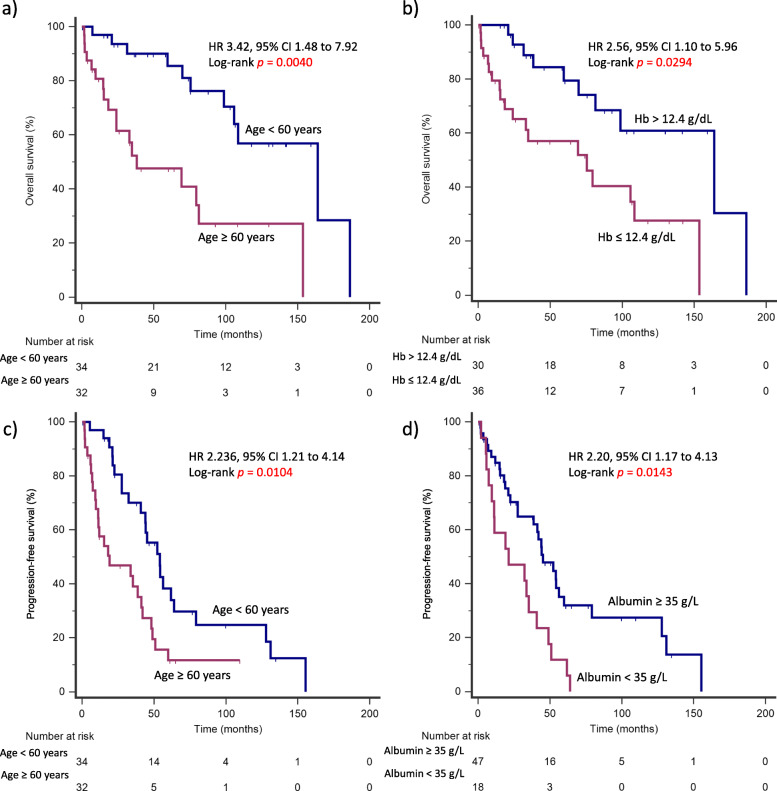
Prognostic factors for survival outcomes in patients with MCL. **a** Independent predictors of worse OS at the time of diagnosis include older age and anemia. **b** Independent predictors of poorer PFS include older age and hypoalbuminemia

At the time of data analysis, 22 patients (33.3%) had died. The 5-year OS and PFS of the global series was 68 and 25.3% respectively. Median OS and PFS were 105.7 months and 41.1 months, respectively (Fig. 1). In univariate analysis, age ≥ 60 years (HR 4.30, 95% CI 1.92–9.63, *p* = 0.0004), ECOG status 1–4 (HR 2.53, 95% CI 1.12–5.71, *p* = 0.026) and Hb ≤ 12.4 g/dL (HR 3.05, 95% CI 1.42–6.57, *p* = 0.0044) were significantly correlated with worse OS**.** In terms of PFS, age ≥ 60 years (HR 2.61, 95% CI 1.40–4.87, *p* = 0.0025), Hb ≤ 12.4 g/dL (HR 2.02, 95% CI 1.12–3.64, *p* = 0.19) and albumin < 35 g/L (HR 3.11, 95% CI 1.48–6.51, *p* = 0.0026) were predictive of poorer outcomes (Fig. 2 and Table 4).

**Table 4:** Univariate survival analysis for entire cohort

Characteristic	Overall survival			Progression-free survival		
	HR	95% CI	***p***-value	HR	95% CI	***p***-value
**Age ≥ 60 years**	**4.30**	**1.92 to 9.63**	**0.0004**	**2.61**	**1.40 to 4.87**	**0.0025**
**Sex (male)**	1.00	0.44 to 2.29	0.9994	0.94	0.50 to 1.77	0.8554
**Ethnicity (Chinese)**	0.73	0.29 to 1.87	0.5160	0.86	0.42 to 1.75	0.6750
**Smoking history present**	1.01	0.30 to 3.38	0.9910	0.73	0.30 to 1.80	0.4964
**B-symptoms present**	1.93	0.87 to 4.29	0.1061	1.26	0.68 to 2.34	0.4711
**ECOG 1–4**	**2.53**	**1.12 to 5.71**	**0.0261**	1.84	0.97 to 3.48	0.0608
**Bulky disease > 10 cm**	0.56	0.14 to 2.34	0.4300	0.71	0.26 to 1.96	0.5136
**sMIPI intermediate-high**	1.50	0.68 to 3.32	0.3184	1.43	0.79 to 2.60	0.2423
**Ann Arbor stage 3–4**	0.60	0.21 to 1.72	0.3398	1.22	0.55 to 2.72	0.6314
**Lymph node involvement ≥ 4**	0.47	0.21 to 1.03	0.0598	0.99	0.55 to 1.78	0.9649
**Extra-nodal involvement**	0.52	0.17 to 1.56	0.2454	0.73	0.31 to 1.70	0.4621
**Bone marrow involvement**	0.46	0.20 to 1.03	0.0586	0.59	0.32 to 1.11	0.1029
**Spleen involvement**	0.50	0.22 to 1.16	0.1081	0.91	0.48 to 1.73	0.7723
**Serum LDH elevated**	1.02	0.46 to 2.27	0.9529	1.59	0.88 to 2.87	0.1240
**Hb ≤ 12.4 g/dL**	**3.05**	**1.42 to 6.57**	**0.0044**	**2.02**	**1.12 to 3.64**	**0.0190**
WBC > 10 * 10^**9**^ **cells/L**	1.54	0.64 to 3.68	0.3311	1.24	0.64 to 2.40	0.5147
**Albumin < 35 g/L**	1.71	0.70 to 4.17	0.2412	**3.11**	**1.48 to 6.51**	**0.0026**

*Abbreviations*: *ECOG* Eastern Cooperative Oncology Group

A multivariate model adjusted for significant clinicopathological parameters for OS and PFS was created. Age ≥ 60 years (HR 3.42, 95% CI 1.48–7.92, *p* = 0.004) and Hb ≤ 12.4 g/dL (HR 2.56, 95% CI 1.10–5.96, *p* = 0.029) were independently associated with poorer OS while age ≥ 60 years (HR 2.24, 95% CI 1.21–4.14, *p* = 0.010) and albumin < 35 g/L (HR 2.20, 95% CI 1.17–4.13, *p* = 0.014) were independently associated with poorer PFS (Table 5).

**Table 5:** Cox’s multivariate survival analysis

Characteristic	Overall survival			Progression-free survival		
	HR	95% CI	***p***-value	HR	95% CI	***p***-value
**Age ≥ 60 years**	**3.42**	**1.48 to 7.92**	**0.0040**	**2.24**	**1.21 to 4.14**	**0.0104**
**Albumin < 35 g/L**	–	–	–	**2.20**	**1.17 to 4.13**	**0.0143**
**Hb ≤ 12.4 g/dL**	**2.56**	**1.10 to 5.96**	**0.0294**	–	–	–

### Lack of survival benefit of maintenance rituximab in older patients

**Fig. 3:**
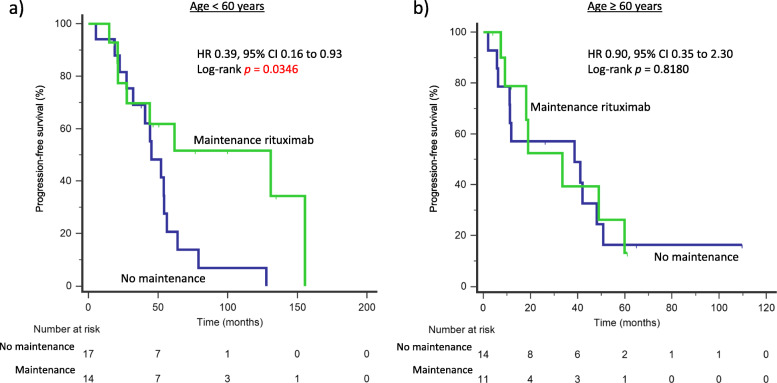
Maintenance rituximab therapy confers PFS benefit in younger but not older patients. **a** Maintenance rituximab improves PFS in patients < 60 years of age following induction chemotherapy, with median PFS of 130.9 months and 45.2 months with or without maintenance therapy, respectively (HR 0.39, 95% CI 0.16–0.93, *p* = 0.0346). **b** No benefit of maintenance rituximab was observed in older patients ≥60 years, with median PFS of 33.5 months and 38.7 months with or without maintenance therapy, respectively

In an exploratory analysis, we demonstrated that MR following induction chemotherapy improved PFS in younger patients (age < 60 years), with median PFS of 130.9 months and 45.2 months with and without maintenance therapy respectively (HR 0.39, 95% CI 0.16–0.93, *p* = 0.0346). In contrast, this benefit was not observed in patients ≥60 years, with median PFS of 33.5 months and 38.7 months with and without maintenance therapy, respectively (Fig. 3). MR conferred a non-statistically significant improvement in OS in patients < 60 years, with median OS being 163.8 months and 105.7 months with and without maintenance therapy, respectively (HR 0.36, 95% CI 0.10–1.26, *p* = 0.1097). For patients ≥60 years, there was no benefit of MR observed on OS, with median OS being 79.4 months and 69.2 months with and without maintenance therapy, respectively (Fig. S1).

## Discussion

Contemporary frontline treatment of MCL involves the use of one of several multimodality immunochemotherapy regimens, with intensity modulated against age and fitness of the patient. Younger and fit patients typically receive rituximab and cytarabine-based induction, often followed by high-dose chemotherapy and auto-SCT [5, 6]. Such intensive regimens are unsuitable for the majority of patients who are older and less fit, though they still benefit from rituximab-containing, non-cytarabine-based chemotherapy such as R-CHOP [8], R-bendamustine [10] or VRCAP [11], as well as regimens containing lower doses of cytarabine such as R-BAC500 [12]. In keeping with this approach, our cohort of MCL patients were treated in a similar fashion, with older patients being treated more often with non-cytarabine-based induction chemotherapy. As demonstrated in our current study and others, these older patients may harbor poor clinical characteristics and prognostic indicators, including worse performance status, B-symptoms, anaemia and high sMIPI scores [13]. Reflecting the issues above, older patients with MCL remain challenging to manage given their adverse clinical features and inability to derive benefit from intensive treatment regimens.

MR following induction therapy forms the current standard of care in several subtypes of non-Hodgkin lymphoma (NHL), including MCL [14, 15]*.* In the ECOG-ACRIN Cancer Research Group study (E1496), MR prolonged PFS when compared to observation alone in patients with indolent NHL after first-line induction chemotherapy [16]. In line with this result, the PRIMA study showed that MR significantly prolonged PFS without an improvement in OS in patients with previously untreated follicular lymphoma [17]. In MCL, an early phase II pilot study by the Wisconsin Oncology Network showed that 2 years of MR may prolong PFS following a modified R-HyperCVAD first-line induction regimen [18]. A major PFS benefit was observed as well compared to observation in patients with relapsed follicular and MCL following salvage chemotherapy [19]. More recent studies have demonstrated significant survival benefit for 3 years of MR in terms of both PFS and OS in younger patients following R-DHAP induction followed by autologous stem cell transplant [20]. In older patients aged 60 years or older, the European MCL Network trial demonstrated that R-CHOP induction followed by MR until progression derived significantly improved PFS and OS benefit compared to maintenance interferon [8]. The benefit of MR in older patients however, appeared to depend on the type of induction used – MR followed by R-FC resulted in high incidence of death in remission, mainly due to infections or secondary tumors, greatly limiting its PFS benefit [8]. In the Stil NHL7–2008 MAINTAIN trial, MR following R-bendamustine induction in a cohort of older MCL patients (median age 70 years) did not lead to significant survival benefit [9]. In our cohort, most of the patients received R-CHOP or cytarabine-based induction prior to MR, though PFS benefit was only observed in younger patients. Taken together, our results support the use of MR after R-CHOP or cytarabine-based first-line induction for younger patients.

Our current study is limited by its retrospective design and small patient cohort. Some of the information relating to prognosis such as immunohistochemical markers and molecular indicators were not available [21], and multivariate analysis may have missed some confounding factors that were not accounted for. The differences observed in baseline characteristics between older and younger patients are also preliminary and remain to be validated. In addition, the treatment received by the patients were heterogeneous, which may have affected their prognosis. Nonetheless, our study remains one of the few to describe the real-world outcomes of MCL in Asian patients. Future prospective studies in a larger cohort would be necessary to confirm our findings.

In conclusion, our study suggests that older patients with MCL may harbor adverse clinical features and may not derive benefit from maintenance rituximab, highlighting the necessity for further work in this area of unmet clinical need.

## Supplementary Information

**Additional file 1: Supplemental Figure S1.** Maintenance rituximab therapy and OS outcomes. (a) Maintenance rituximab in patients < 60 years of age following induction chemotherapy confers a non-statistically significant improvement in OS compared to those without maintenance therapy (median OS 163.8 months and 105.7 months, respectively) (HR 0.36, 95% CI 0.10–1.26, *p* = 0.1097). (b) No benefit of maintenance rituximab was observed in older patients ≥60 years, with median OS of 79.4 months and 69.2 months with or without maintenance therapy, respectively.

**Additional file 2: Supplemental Table S1**. Clinical and demographic characteristics of patients receiving chemotherapy.

## Data Availability

The datasets created and analysed during this study are available from the corresponding authors upon reasonable request.
