# Supplementary material for: Treatment patterns and outcomes of older patients with mantle cell lymphoma in an Asian population
Source: BMC Cancer. 2021 May 17;21:566. doi: 10.1186/s12885-021-08326-1 (PMC8130422; doi:10.1186/s12885-021-08326-1)

**Supplemental Figure S1. Maintenance rituximab therapy and OS outcomes.** (a) Maintenance rituximab in patients < 60 years of age following induction chemotherapy confers a non-statistically significant improvement in OS compared to those without maintenance therapy (median OS 163.8 months and 105.7 months, respectively) (HR 0.36, 95% CI 0.10-1.26, *p* = 0.1097). (b) No benefit of maintenance rituximab was observed in older patients ≥ 60 years, with median OS of 79.4 months and 69.2 months with or without maintenance therapy, respectively.


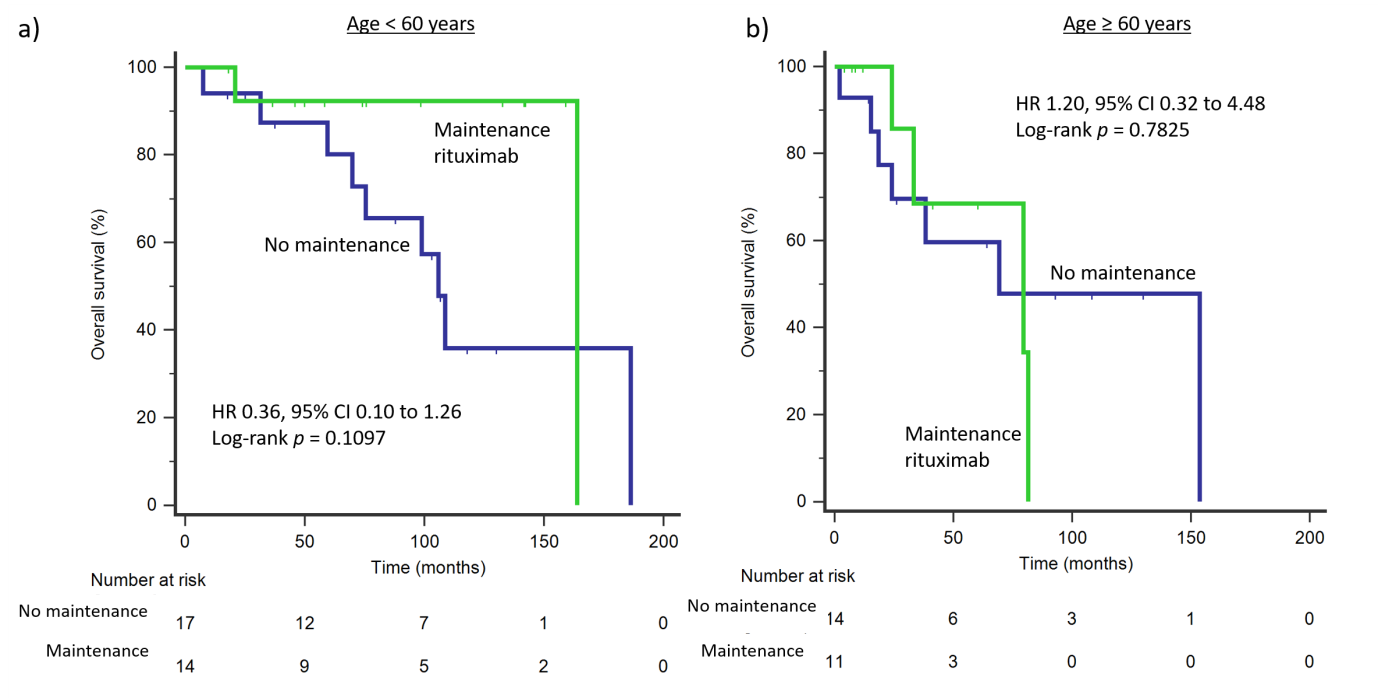

Supplement: Supplementary file 1 — Additional file 1: Supplemental Figure S1. Maintenance rituximab therapy and OS outcomes. (a) Maintenance rituximab in patients < 60 years of age following induction chemotherapy confers a non-statistically significant improvement in OS compared to those without maintenance therapy (median OS 163.8 months and 105.7 months, respectively) (HR 0.36, 95% CI 0.10–1.26, p = 0.1097). (b) No benefit of maintenance rituximab was observed in older patients ≥60 years, with median OS of 79.4 months and 69.2 months with or without maintenance therapy, respectively. [file 12885_2021_8326_MOESM1_ESM.docx]
