# Supplementary material for: Treatment patterns and outcomes of older patients with mantle cell lymphoma in an Asian population
Source: BMC Cancer. 2021 May 17;21:566. doi: 10.1186/s12885-021-08326-1 (PMC8130422; doi:10.1186/s12885-021-08326-1)
Supplement: Supplementary file 2 — Additional file 2: Supplemental Table S1. Clinical and demographic characteristics of patients receiving chemotherapy. [file 12885_2021_8326_MOESM2_ESM.docx]

**Supplemental Table S1. Clinical and demographic characteristics of patients receiving chemotherapy**

| **Characteristic** | **Age at diagnosis (years)** | | ***p*-value** |
| --- | --- | --- | --- |
|  | **≥ 60** | **< 60** |  |
| ***Total*** (56) | 25 (44.6%) | 31 (55.4%) | - |
| ***Sex*** | | | |
| **Male** (41) | 20 (80%) | 21 (67.8%) | 0.3720 |
| **Female** (15) | 5 (20%) | 10 (32.2%) |  |
| **Ethnicity** |  |  |  |
| **Chinese** (43) | 18 (72%) | 25 (80.6%) | 0.4503 |
| **Other** (13) | 7 (28%) | 6 (19.4%) |  |
| ***Smoking history*** |  |  |  |
| **Yes** (6) | 3 (12%) | 3 (9.68%) | 1.0000 |
| **No** (50) | 22 (88%) | 28 (90.3%) |  |
| ***B-symptoms*** | |  |  |
| **Present** (17) | 5 (20%) | 12 (38.7%) | 0.1548 |
| **Absent** (39) | 20 (80%) | 19 (61.3%) |  |
| ***ECOG performance status*** | |  |  |
| **0** (19) | 10 (40%) | 9 (29.0%) | 0.3931 |
| **1-4** (37) | 15 (60%) | 22 (71.0%) |  |
| ***Ann Arbor Stage*** | |  |  |
| **1-2** (5) | 1 (4%) | 4 (12.9%) | 0.3670 |
| **3-4** (51) | 24 (96%) | 27 (87.1%) |  |
| ***sMIPI risk*** |  |  |  |
| **Low/intermediate** (18) | 4 (16%) | 14 (45.2%) | **0.0244** |
| **High** (38) | 21 (84%) | 17 (54.8%) |  |
| ***Bulky disease > 10cm*** | |  |  |
| **Yes** (6) | 3 (12%) | 3 (9.68%) | 1.0000 |
| **No** (47) | 21 (84%) | 26 (83.9%) |  |
| ***Spleen involved*** | |  |  |
| **Yes** (18) | 9 (36%) | 9 (29.0%) | 0.5823 |
| **No** (38) | 16 (64%) | 22 (71.0%) |  |
| ***Number of nodal sites*** | |  |  |
| **0-3** (19) | 6 (24%) | 13 (41.9%) | 0.1625 |
| **≥ 4** (37) | 19 (76%) | 18 (58.1%) |  |
| ***Extra-nodal involvement*** | |  |  |
| **Yes** (47) | 19 (76%) | 28 (90.3%) | 0.2721 |
| **No** (9) | 6 (24%) | 3 (9.68%) |  |
| ***Bone marrow involvement*** | |  |  |
| **Positive**  (38) | 15 (60%) | 23 (74.2%) | 0.2625 |
| **Negative** (18) | 10 (40%) | 8 (25.8%) |  |
| ***Ki-67 expression (%)*** |  |  |  |
| **> 30** (19) | 9 (36%) | 10 (32.4%) | 1.0000 |
| **≤ 30** (22) | 10 (40%) | 12 (38.7%) |  |
| ***Serum LDH*** |  |  |  |
| **Elevated** (30) | 14 (56%) | 16 (51.6%) | 0.6228 |
| **Not elevated** (25) | 10 (40%) | 15 (48.4%) |  |
| ***Albumin (g/L)*** |  |  |  |
| **< 35** (15) | 9 (36%) | 6 (19.4%) | 0.1376 |
| **≥ 35** (40) | 15 (60%) | 25 (80.6%) |  |
| ***Hemoglobin (g/dL)*** |  |  |  |
| **≤ 12.4** (28) | 17 (68%) | 11 (35.5%) | **0.0165** |
| **> 12.4** (28) | 8 (32%) | 20 (64.5%) |  |
| ***WBC (10^9^ cells/L)*** |  |  |  |
| **> 10** (15) | 6 (24%) | 8 (25.8%) | 0.8778 |
| **≤ 10** (41) | 19 (76%) | 23 (74.2%) |  |
| **Induction chemotherapy^a^** |  |  |  |
| **Cytarabine-based** (27) | 6 (24%) | 21 (67.7%) | **0.0012** |
| **Other** (29) | 19 (76%) | 10 (32.3%) |  |
| **Maintenance Rituximab** |  |  |  |
| **Yes** (25) | 11 (44%) | 14 (45.2%) | 0.9314 |
| **No** (31) | 14 (56%) | 17 (54.8%) |  |

Variables unknown include: presence of bulky disease > 10 cm (n = 3), Ki-67 expression (n = 15), serum LDH (n = 1)

**^a^**For age ≥ 60: R-HyperCVAD (n=2), R-CHOP/R-AraC (n=2), R-BAC (n=2), R-CHOP (n=10), CHOP (n=2), R-CVP (n=1), VRCAP (n=2), R-bendamustine (n=4); for age < 60: R-HyperCVAD (n=14), HyperCVAD (n=2), R-CHOP/R-AraC (n=4), R-CHOP/R-DHAP (n=1), R-CHOP (n=6), VRCAP (n=1), R-bendamustine (n=3)
